# A Neural Computational Model of Incentive Salience

**DOI:** 10.1371/journal.pcbi.1000437

**Published:** 2009-07-17

**Authors:** Jun Zhang, Kent C. Berridge, Amy J. Tindell, Kyle S. Smith, J. Wayne Aldridge

**Affiliations:** 1 Department of Psychology, University of Michigan, Ann Arbor, Michigan, United States of America; 2 McGovern Institute for Brain Research, Massachusetts Institute of Technology, Cambridge, Massachusetts, United States of America; University College London, United Kingdom

## Abstract

Incentive salience is a motivational property with ‘magnet-like’ qualities. When attributed to reward-predicting stimuli (cues), incentive salience triggers a pulse of ‘wanting’ and an individual is pulled toward the cues and reward. A key computational question is how incentive salience is generated during a cue re-encounter, which combines both learning and the state of limbic brain mechanisms. Learning processes, such as temporal-difference models, provide one way for stimuli to acquire cached predictive values of rewards. However, empirical data show that subsequent incentive values are also modulated on the fly by dynamic fluctuation in physiological states, altering cached values in ways requiring additional motivation mechanisms. Dynamic modulation of incentive salience for a Pavlovian conditioned stimulus (CS or cue) occurs during certain states, without necessarily requiring (re)learning about the cue. In some cases, dynamic modulation of cue value occurs during states that are quite novel, never having been experienced before, and even prior to experience of the associated unconditioned reward in the new state. Such cases can include novel drug-induced mesolimbic activation and addictive incentive-sensitization, as well as natural appetite states such as salt appetite. Dynamic enhancement specifically raises the incentive salience of an appropriate CS, without necessarily changing that of other CSs. Here we suggest a new computational model that modulates incentive salience by integrating changing physiological states with prior learning. We support the model with behavioral and neurobiological data from empirical tests that demonstrate dynamic elevations in cue-triggered motivation (involving natural salt appetite, and drug-induced intoxication and sensitization). Our data call for a dynamic model of incentive salience, such as presented here. Computational models can adequately capture fluctuations in cue-triggered ‘wanting’ only by incorporating modulation of previously learned values by natural appetite and addiction-related states.

## Author Summary

Reward cues are potent triggers of desires, ranging from normal appetites to compulsive addictions. Food cues may trigger a sudden desire to eat before lunch, and drug cues may trigger even a ‘recovered addict’ to relapse again into drug taking. But learned cues are not constant in their motivating power. Food cues are more potent when you are hungry, and drug cues may become overwhelmingly potent to an addict who tries to take ‘just one’ drink or hit, precipitating an escalating binge of further relapse. These changes in cue-triggered desire produced by a change in biological state present a challenge to many current computational models of motivation. Such modulation can even be unlearned (though the modulation interacts with cues acquired through learning), in that the modulation instantly follows a physiological or neurobiological change (hunger, drug hit, etc.), altering the cue's ability to trigger desire for a relevant reward. Here we demonstrate concrete examples of instant modulation and propose how to build computational models of cue-triggered ‘wanting’ to better capture the dynamic interaction between learning and physiology that controls the incentive salience mechanism of motivation for rewards.

## Introduction

Incentive salience [1],[2] is a mechanism to explain motivational values of specific learned stimuli (Pavlovian conditioned stimuli) and associated natural rewards (unconditioned stimuli) in humans and animals [3]–[5]. The incentive salience framework postulates a fundamental dissociation in brain mechanisms between reward ‘liking’ and reward ‘wanting’. ‘Liking’ is the hedonic impact or pleasure associated with the receipt of an immediate reward while ‘wanting’ is incentive salience itself: a motivational magnet quality that makes the conditioned or unconditioned stimulus a desirable and attractive goal [6],[7]. Psychological incentive salience is actively attributed by brain systems to a sensory stimulus, transforming it from a mere sensory representation into a ‘wanted’ and attractive incentive capable of grabbing attention and motivating approach, seeking and consumption behaviors. Although nonhedonic, unlike ‘liking’, which reflects the pleasure or hedonic impact of the stimulus, incentive salience (‘wanting’) is still motivational.

The incentive salience of a cue is established by learning Pavlovian S-S associations between a cue (conditioned stimulus or CS) and its reward (unconditioned stimulus or UCS). However, the incentive salience framework also postulates a difference between learning and ‘wanting’: especially evident as post-learning dissociations between the previously learned values of a stimulus and its motivational value at a later moment [1], [2], [7]–[9]. Incentive salience can dynamically change, being generated afresh each time the stimulus is re-encountered and incorporating into the computation a second source of input besides previously-learned cached values. This second source of input is physiological state, which includes natural hunger or thirst appetite and satiety states, drug-induced states such as intoxication priming, withdrawal and permanent sensitization, and states of brain mesocorticolimbic systems (often involving dopamine) translate these physiological states into motivation. In this paper we focus on the dynamic nature of this interaction between learned and physiological inputs that combine to modulate motivation.

Importantly, physiological modulation of cue-triggered incentive salience can occur immediately when a state changes, without necessarily any need for additional learning. The most convincing example occurs when a never-before experienced physiological state, such as a specific appetite, modulates cue-triggered motivation appropriately for a previously learned Pavlovian cue [2], [3], [9]–[14]. Natural specific appetites, such as salt appetite, dynamically and appropriately modulate incentive salience or ‘wanting’ triggered by their own reward cues. Conditioned stimuli associated with salt, food, or drink rewards [3],[5] are thus modulated in incentive salience value (in parallel with the alliesthesia modulation of the hedonic value of their UCS rewards, but perhaps even more strongly [15]).

The underlying physiological mechanism of incentive salience is postulated to involve mesocorticolimbic brain systems that involve dopamine activation [2]. The relationship to brain dopamine states makes incentive salience vulnerable to enhancement by psychostimulant drugs that activate dopamine systems. Enduring neural sensitization of dopamine-related mesolimbic systems induced by repeated exposures to drugs is the basis for the incentive-sensitization theory of drug addiction [7]. Sensitization of dopamine-related systems by binging on drugs such as heroin and cocaine is thought to produce long-lasting hyper-excitability of the brain's mesocorticolimbic system and to result in compulsive ‘wanting’ to take drugs and in cue-triggered relapse. Sensitized ‘wanting’ may effectively trigger relapse even when addicts may not derive much pleasure from the drugs, nor expect to derive much pleasure, and even long after the addict is free of withdrawal symptoms.

One implication of the dependence of incentive salience on both previously-learned Pavlovian associations and the current physiological state is that the motivation values of learned cues may vary from what was previously learned. The incentive value of a cue will equal the learned value *if and only if* the physiological state during testing is similar to the state during learning. When physiological states shift, the motivational value may be transformed from the learned value, either up or down, even if the cue and reward have not yet been experienced in the new state. The new ‘wanting’ value is revealed when the cue is re-encountered again. Thus, incentive salience maps onto *decision utility* triggered by a reward cue. This decision utility can be changed by shifts of physiological state without necessarily changing either the *remembered utility* of the reward previously associated with that cue, the *predicted utility* of expected future rewards, or the hedonic *experienced utility* of those rewards when eventually consumed [2],[9],[16].

The distinction between decision utility and remembered/predicted utility places a firewall between motivation and learning that protects the integrity of learned values. Cached values for Pavlovian associations do not need to be rewritten in order to explain changes in incentive salience. Further, cognitive predictions of future value need not be distorted by fluctuating appetite states.

At the same time, the firewall allows the incentive salience of the same Pavlovian cues to be more labile, and to change more in lockstep with relevant physiological shifts [17],[18]. A consequence is that an individual can have a change in ‘wanting’ without necessarily changing expectations of future liking, as well as without changing actual ‘liking’ of the reward when it occurs. Thus, incentive salience may sometimes be magnified to the point that produces ‘irrational wants’ where decision utility is strongly elevated over the concomitant predicted utility. This mismatch can have a dire outcome, such as when drug addicts who have unwittingly caused mesolimbic sensitization in their own brains may persist irrationally in cue-triggered pulses of ‘wanting’ to take drugs, even when they recognize cognitively that the drug may not be very pleasant, not worth the costs, and they wish to abstain [7]–[9]. Such elevations in ‘wanting’ can occur without necessarily any fluctuation in learned associations or in cognitive predictions of reward value.

### Incentive salience and dopamine prediction error models of reward learning

Incentive salience is essentially a Pavlovian motivational response: it takes Pavlovian associations as its learned input. Potential differences between cached valence and motivation values for Pavlovian cues have been previously noted by computational modelers and learning theorists [14], [18]–[22]. For example, Dayan and Balleine noted that, “Pavlovian CRs are … directly sensitive to the level of motivation of the subjects, such as whether or not they are hungry. This motivational or affective control argues against simple realizations of the critic in which there is one set of weights or parameters (whatever their neural realization) mapping from stimulus representations (i.e., units whose activities are determined by the stimuli shown to the subject) to the values” (p. 288, [14]).

Here we aim to account for such motivation variations by identifying incentive salience computations that dynamically determine Pavlovian motivation value. These computations must integrate Pavlovian learned inputs with the status of the brain mesolimbic mechanisms that reflect current physiological states (hungers, drug intoxication, sensitization, etc.).

To see the difference between the vantage points of incentive salience compared to reward learning, it may be helpful to review how cached values are established in contemporary reinforcement learning models. For example, the temporal-difference (TD) method provides an explicit “model-free” formula for estimating expected reward. It is model-free in the sense that it does not involve an internal model or cognitive map of the world, but depends only on cached experiences and the accumulated state for estimating the value function. The estimate is based on summed values from reward prediction errors – the discrepancy between the reward expected from a stimulus (technically, a state) and the reward actually received [20], [23]–[29]. One influential neurobiological view identifies the predictive error signal, which lies at the core of temporal difference learning, with the firing of dopaminergic neurons projecting to the nucleus accumbens and neostriatum [30],[31] (though this notion is not without controversy regarding the causal role of dopamine systems in generating prediction errors and value estimates [2],[32]). The actor-critic architecture, along with the TD-based learning rule, carries great computational power. It provides, at least theoretically, a consistent and effective scheme to solve the so-called “dynamic programming” problem [33] concerning optimization for sequential decision-making under a stationary Markov environment, without the need of an elaborate model of the world (i.e., how states unfold successively and how one's actions affect such state-transition) [27], [31], [34]–[36].

The TD error model also has been applied to incentive salience [26]. A first step was taken by McClure, Daw and Montague, who used the concept of prediction error-driven learning, and equated cue-triggered ‘wanting’ with the cached Pavlovian learned value acquired by the TD method [26]. Their model postulated incentive salience to function as a cached, cumulative reward value, which, if a reward was suddenly revalued, could be changed only by further relearning about a new prediction error by re-encountering the UCS. They suggested that “the role of dopamine in learning to attribute such expectations to situations that are predictive of reward and in biasing action selection towards such situations as the formal counterpart to the ideas of Berridge and Robinson about the role of dopamine in attributing and using incentive salience.” p. 425, [26]. While a useful contribution that exploited the TD model's strengths to capture trial-by-trial ‘reboosting’ of ‘wanting’, we believe it is only an initial step towards modeling incentive salience.

Despite the computational success of actor-critic architecture, several theorists have suggested, as noted above and consistent with our view here, that additional mechanisms must be added to explain emerging psychological and neurobiological data and to account for motivation because the simple form of actor-critic architecture produces a rigidly incremental cached value of prediction [14],[37],[38]. One solution to account for immediate state-based changes in behavior has been to posit that dopamine also modulates the “vigor” of all responses in a general fashion [37],[38]. Additionally, uncertainty or generalization decrements after a state change has been suggested to explain some reduction in behavioral responses, at least for changes that devalue a reward downwards [39]. Another alternative solution is to sidestep the model-free cached limitations and add an entirely separate model-based (e.g., cortically-embedded) mechanism for reward prediction in the form of a model-based or tree-search system that explicitly represents the world as a cognitive “state space”. Such a cognitive model includes goal values and act-outcome (A-O) relationships, and which can adjust instrumental behavior more flexibly, at least once a new goal value is known by experience [14], [17], [18], [21], [22], [39]–[41].

Still, cue-triggered ‘wanting’ differs from all the above, and we believe may more accurately capture the chief motivation function of brain mesocorticolimbic systems. The re-computations of the incentive salience for a Pavlovian CS may in some cases be carried out in a highly dynamic, stimulus-specific and stimulus-bound fashion, as will be described below. This is possible because of Bindra-Toates rules of Pavlovian motivation that underlie incentive salience [3]–[5]. Those rules are distinct from both cached TD values and model-based cognitive predictions.

Cached values and model-based predictions of reward both often assume that a reward will be about as good in the future as it was in the past [14],[22],[26],[40]. Robust computational theories exist for those cached model-free values and cognitive model-based systems, but not yet for Bindra-Toates computations of incentive salience. A similar sentiment was recently expressed by Dayan and Niv, “Unfortunately, the sophisticated behavioral and neural analyses of model-free and model-based instrumental values are not paralleled, as of yet, by an equivalently worked-out theory for the construction of Pavlovian values.” (p. 191) [22].

Our goal here is to take a small step towards a better computational theory for Pavlovian-guided generation of incentive salience. Specifically, we aim for a model able to compute cue-triggered ‘wanting’ even for novel physiological modulations that occur before there is an opportunity for relearning the altered reward. We also aim for a model that can account for ‘irrational wanting’ in addictions; that is, for excessively ‘wanting’ a reward even when knowing its future value to not deserve intense motivation.

Consistent with contemporary views, we believe that multiple reward-related learning processes exist within a single brain, mediated by separable brain systems [7], [14], [17], [18], [21], [22], [38]–[40], [42]–[45]. We presume these reward learning-motivation mechanisms occur in parallel as three separable processes (S-S Pavlovian-guided incentive salience, S-R cached habits, and model-based cognitive expectations). Our model is meant to capture only incentive salience transformations, which takes Pavlovian CS-UCS associations involving rewards as the primary learned input for Bindra-Toates modulations.

### Incentive salience and dynamic shifts of value

Our view of incentive salience calls for the dynamic computation of “incentive value” of a conditioned or unconditioned stimulus, where (a) the CS stimulus has previously been associated with the relevant UCS; and (b) the value is gain-controlled moment to moment by fluctuations in relevant physiological states (including neurobiological states of brain mesocorticolimbic systems).

Recall that in reinforcement learning, the expected total future discounted reward (or simply average reward value) *V* associated with a state *s* (i.e., the conditioned stimulus) is
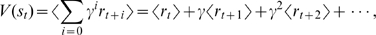
(1)where 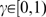 is the discount factor, *r_t_*, *r_t+1_*, *r_t+2_* …, representing the sequence of primary rewards starting from the current state (subscripted *t*), and the expectation 〈·〉 is taken over possible randomness in environmental state transition and reward delivery (the bracket sign around primary reward values will be omitted below for clarity). The estimated value of reward prediction 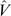 (denoted with a hat) is a value gradually acquired by the agent through temporal difference learning over a series of experiences in which the predictive CS and UCS reward are paired. The acquisition of reward estimate 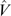 is based on computing a prediction error *δ* correcting any experienced deviation from consistent successive predictions:
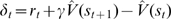
(2)and then updating 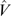 according to 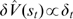. The value function *V* is essentially an incrementally-learned associative prediction of each state. As mentioned, one previous computational proposal for incentive salience equated this value function *V*, defined by Eqn (1), with the motivational concept of CS incentive salience [26], and gradually altered motivational value by increments in *V* in each trial produced by prediction error at the moment when reward UCS was received, via the temporal difference error variable *δ*, defined by Eqn (2). The TD error *δ* was identified with the “reboosting” process of a CS posited by the incentive salience hypothesis to occur at the moment of UCS [1],[46]. Reboosting was a concept added a decade earlier by the original incentive salience proposals to account for the gradual decrement effects on rewarded behavior produced by administering neuroleptic drugs that partially blocked dopamine receptors (anhedonia-like effects or extinction mimicry) [47]. In such a conceptualization, the incentive salience of a stimulus is essentially the accumulated reinforcement value of such a conditioned stimulus acquired through TD prediction-error learning.

## Methods

### Ethics statement

All animal work was conducted according to relevant University of Michigan, NIH, national and international guidelines.

### A computational model for incentive salience: κ factor for dynamic physiological shifts

We suggest additionally that a model of incentive salience should also incorporate dynamic physiological modulation by current states to capture more sudden changes in CS motivational value, such as in specific appetites or drug enhancement of ‘wanting’, which do not proceed by gradual reboosting via UCS prediction errors [1],[2],[48],[49]. In these conditions, a CS's motivationally-transformed incentive salience value may dramatically diverge from cache-generated predictions of reward [10], [12]–[14],[40],[44],[50]. In at least some cases these physiological revisions of value can occur without any need of relearning about the change in UCS hedonic impact to revise CS-UCS predictions, yet still be so powerful as to completely reverse the incentive value of a CS.

In the following, we will first propose a model for incentive salience that can incorporate dynamic modulation of cue-triggered ‘wanting’ by even novel physiological states. Next, we will show how such a model maps onto empirical evidence for dynamic modulation in examples of natural appetite, amphetamine intoxication, and addiction-related sensitization.

To describe our model of incentive salience more precisely, here we propose that, respecting the difference between motivation and learning, *incentive salience computations incorporate a physiological factor κ that modulates the value of a CS associated with a relevant UCS (which carries a reward value of r_t_)*. The κ factor reflects current physiological state (hungers, satieties, drug states, etc.). The role of κ is to allow incentive salience of an associated CS to be dynamically modulated by physiological factors relevant to future rewards (e.g., hungers, satiety, drug intoxication, mesolimbic sensitization, etc.).

### Model

We suggest that the incentive salience or motivational value 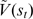 of the current state in the presence of a reward CS is
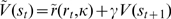
(3)Equation (3) represents a generic model for incorporating the motivation factor *κ* which modulates the learned representation of primary reward and hence the notion of incentive salience, where *κ* is a positive constant that varies with behavioral state. Here 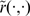 is a generic two variable function which, in the following discussions, will be specialized to either of two forms (sub-types), as described below.
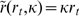
(3a)

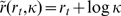
(3b)


Below, we refer to *κ* as the “gating parameter” for incentive computations involving physiological manipulations for a previously learned CS (e.g. hunger, thirst, salt appetite, etc.), with *κ*<1 representing devaluation (such as satiation), and *κ*>1 representing enhancement (such as appetite or sensitization). When *κ* = 1 our model reduces to the conventional temporal difference model; that is, when physiological state remains constant across training and test.

**Figure 1:**
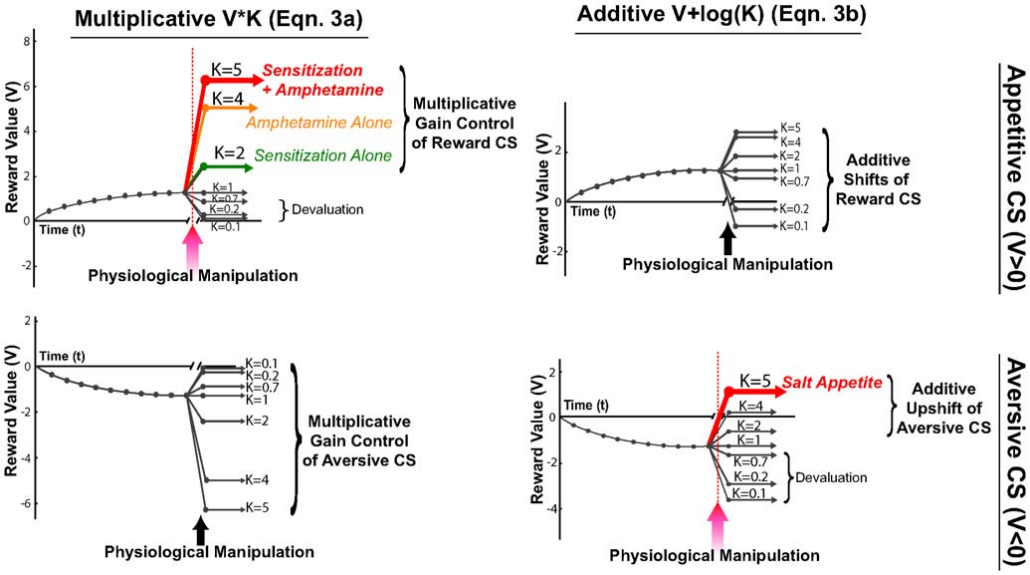
Simulations of dynamic shifts in incentive salience. All are induced by changes in physiological state after learning a CS-UCS association in an initial state (Eqn 3). The left column is for multiplicative mechanisms (Eqn 3a), while the right column is for additive mechanisms (Eqn 3b). The top row is for shifts after learning a Pavlovian association with a reward UCS (e.g., sucrose taste), and the bottom row is for shifts after learning with an aversive UCS (e.g., intense salt taste). Initial learning is assumed to proceed by a Rescorla-Wagner type of rule initially [20] (t = 0,1,…,10) as described by the equation V(t) = A (1−exp (−t/τ)), with asymptote A = 1.3 for appetitive reward and A = −1.3 for aversive reward, and the time constant τ = 3. At time step t = 11, a new motivation manipulation is introduced, such as by a shift in a physiological state relevant to the reward. The change in incentive salience occurs as indicated by the arrows, either multiplicatively (V*κ) or additively (V+log κ), where κ (for illustrative purpose) takes the values 5,4,2,1, 0.7, 0.2, 0.1. See Eqn (3a,3b) of the manuscript. Colored lines in the upper-left panel describes Experiment 2 (drug sensitization and acute amphetamine administration), while the lower-right panel depicts Experiment 1 (salt appetite). Note that additive modulation can reverse reward valence, while multiplicative modulation maintains the original reward valence and changes only magnitude.

When physiological state changes from training to test, one of the two special versions of Equation 3 will apply, and which of the two is most appropriate will depend on the situation (Figure 1). Equation (3a) describes a specific subtype in the form of a multiplicative mechanism, appropriate for most situations where motivation changes from low to high or vice versa without changing valence — reward is manipulated between 0 and a positive value, changing incentive salience from neutral to ‘wanted’ (or from ‘wanted’ to neutral). In these cases, *κ* is a gain-control factor that scales up (i.e., magnifies, when *κ*>1) or down (i.e., shrinks, when *κ*<1) the incentive salience of the reward.

Equation (3b) describes another subtype in the form of an additive mechanism, appropriate for other situations where incentive value not only changes but reverses valence between positive and negative — this can additionally account for any special cases in which a reward value changes polarity from positive to negative or from negative to positive. In those cases, the log*κ* term moves the baseline of the incentive salience value, which can be shifted either up (i.e., increases ‘wanting’, *κ*>1) or down (i.e., decrease ‘wanting’, *κ*<1). This allows polarity reversal from a negative value to a positive value (with *κ* much larger than 1), or vice versa (with *κ* closer to 0).

The reason why we include both an additive and a multiplicative version of modulation in Equation (3) is to more sensibly achieve real-life reversals than can be accomplished in a purely multiplicative model by simply changing the *κ* valence polarity to negative. This is because merely changing polarity in a multiplicative Equation (3a) would invert the rank order when multiple reward stimuli in the same family were involved (e.g. 3 concentrations of salt). That would revalue the respective order of the series in ways that might be unrealistic. For example, reversing valence in a multiplicative model would cause the reward that was originally most highly liked and ‘wanted’ of all to become the most highly disliked or repulsive after devaluation of all; an intermediately liked reward would become intermediately disliked, while a nearly neutral reward stimulus would remain nearly neutral after polarity reversal. Such re-ordering fails to describe what happens in empirical cases of valence reversal, where the originally most liked reward may often still remain the best of a bad lot, becoming the least disliked as a physiological manipulation changes the valence of the entire group.

By contrast, an additive model as expressed in Equation (3b), allows the ‘best’ stimulus to remain best relative to the others, even if their absolute values may switch valence (i.e., all shift across zero). Specific candidates for polarity reversal include reversals in reward values from nasty to nice, such as described below where an intensely salty taste reward is re-encountered during a salt appetite, or from nice to nasty such as after taste-aversion devaluation [10], [50]–[52] (Figure 1). Polarity reversal would similarly encompass cases in which motivational salience changes valence between desire and dread [53],[54].

We remark in passing that we use log*κ* instead of *κ* for the additive Eqn (3b) simply to have the same parametric representation in the additive case as the multiplicative case. Also note that we only consider additive and multiplicative mechanisms which together generate the group of (positive) affine transformations on reward values (this is the class of transformation that keeps the optimal policy invariant [55]).

From Eqns (3) and (1), and assuming a multiplicative mechanism, the incentive value 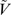 is related to the average reward *V* (i.e., total reward including current and all future-discounted rewards) via
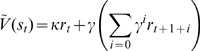
(4)


In essence, Eqn. (4) is an expression of what is known as the “quasi-hyperbolic” discounting model encountered in economics literature [56],[57]. The proposal here exploits the two parameters in the quasi-hyperbolic discount model format and is the basis of our current postulate of a gain-control mechanism to implement incentive salience computations, in a manner that can be sensitive to inter-temporal comparisons of values when such comparisons play important roles [58], cf. [59]. In simple terms, our model of incentive salience 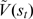 reduces to 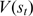 in the absence of devaluation/sensitization manipulation (*κ* = 1). The modulatory factor *κ* is assumed to be independently controlled by the geometric temporal discounting under *γ*, though it is possible that such changes can be coupled. For example, sensitization or an increased physiological appetitive state (*κ* becomes greater than 1) might lead to a decrease in the temporal horizon *γ* [60], producing sharper temporal discounting effects, such that motivational value increases with degree of temporal proximity to reward UCS [61]. Note that the incentive value of a state *s_t_* is the motivationally-modulated value 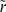 of the immediate reward *r_t_* plus the discounted value of the expected reward in the next state *s_t+1_*; both these are loaded into the goal representation as *s_t_* is presented.

#### Shielding learned values from physiological modulation

One reason for our current model to treat *κ* as a multiplicative or additive parameter is that we wish to strongly distinguish incentive salience as a motivation value which integrates learning and physiological inputs from the stable, purely-learned and cached value *V* per se (or for that matter even *Q* value). Both *V* and *Q* require learning to establish, become cached once learned, and as stable memory values both are shielded from moment-to-moment modulation by an animal's motivational state. By contrast, incentive salience is modeled here with the feature of being able to globally modulate the on-line evaluation of previously-learned values of a primary reward evoked by a CS.

#### Specific κ's determine what to ‘want’

It is important to note we conceive *κ* to be specific to a particular appetite state and to its own relevant UCS reward and CSs. There can be different values of *κ* at any given time for different CSs that are associated with different reward UCSs (food, salt, water, sex etc.). This specificity includes specific natural appetites, such as salt, hunger, thirst, etc; as well as mesolimbic sensitization involved in drug addiction. Each appetitive system would specifically modulate the values of its own UCS array (e.g., salt is the UCS reward for salt appetite) and simultaneously modulate most powerfully specific CSs associated with its UCSs. This specificity of *κ* modulation gives a basis for why drug addicts mostly ‘want’ drugs, binge eaters mostly ‘want’ food, sodium deficient individuals mostly ‘want’ salt, and so on. Still, under some conditions, incentive salience may spill over from one appetite state to modulate CSs for another appetite state. For example, some cocaine addicts may also show a degree of hyper-sexuality, compulsive gamblers may show other addictions, and drug sensitization may sometimes enhance the motivational value of natural rewards (see below).

In sum, we propose that the computational substrate for incentive salience must instantiate 1) *κ*, an online gain or baseline control (collectively called “gating”) mechanism that can be dynamically modulated by changes in physiological or neurobiological states to target motivation towards a relevant CS along with 2) possible adjustment of the temporal horizon *γ* for evaluating stored prediction values. Both substrates are assumed to be adjustable from moment to moment by physiological states, without necessarily requiring new (re)learning.

### Neural bases for incentive salience in mesocorticolimbic circuits

The incentive salience hypothesis specifically proposes that Pavlovian-guided attribution of incentive salience is mediated principally via subcortically-weighted NAcc-related circuitry involving dopamine neurotransmission, which pass signals through the ventral pallidum. These circuits include input from mesolimbic dopamine projections from ventral tegmentum and substantia nigra to the nucleus accumbens, ventral pallidum, and amygdala; and output projections from nucleus accumbens that converge through ventral pallidum [62]–[64]. From ventral pallidum these signals then pass to a thalamic relay for return to mesocorticolimbic loops, or directly descend to other subcortical outputs [62],[65],[66]. In addition to receiving mesolimbic outputs, dopamine projections from VTA also ascend directly to ventral pallidum [67],[68]. Thus the incentive salience hypothesis views incentive salience or ‘wanting’ to be influenced by dopamine-related modulations of function within this circuit, the output of which passes through ventral pallidum as a limbic ‘final common path’.

The computational approach suggested here can therefore be tested empirically by measuring neural signals carrying incentive salience in the final common path through ventral pallidum, in experiments which manipulate NAcc-related circuitry via changes in natural appetite states (hungers, satieties) or via addictive drugs (drug administration; long-term drug sensitization).

## Results

### Empirical tests: natural appetites and addictive drugs

To illustrate our proposal about the computation of incentive salience, we now draw on two types of experiments designed to expose dynamic physiological modulation of cue-triggered ‘wanting’, as posited in equation (3). The first experiment uses the natural motivation state of salt appetite to change the incentive salience of a salt CS. The second experiment uses a dopamine-stimulating drug amphetamine and/or enduring drug-induced sensitization to activate mesolimbic NAcc-related systems and change the incentive salience of a sucrose CS.

#### Test 1: natural appetite states dynamically elevate CS ‘wanting’

The natural homeostatic state of salt appetite is especially useful for probing dynamic shifts in incentive value because this natural appetite can be introduced as a novel experimental state. Novelty is enabled because sodium deficiency is almost never experienced by laboratory animals or modern humans who eat diets that contain plenty of sodium chloride. Salt appetite emerges only in physiological states when sodium is depleted from the body (e.g., caused by drugs or by subsistence on a very low-sodium diet). Salt appetite causes intense elevation in both salt ‘wanting’ and salt ‘liking’ [51],[69]. At the same time, a CS (a sound, sight or a sour or bitter taste) previously associated with the salty taste also is dynamically revised from negative to positive in both incentive salience value and hedonic value [10]–[12],[50],[70].

We set a strict criterion for what must occur if those signals constitute a dynamic enhancement of incentive salience: neural signals for CS value in ventral pallidum signals must dynamically and selectively rise to a salt CS on its first ever presentation in a sodium appetite state, even prior to re-tasting salt itself in the new state. A neurobiological experiment in our lab was designed to test the incentive salience hypothesis as modeled by Eqn (3) [12]. In it, rats in a normal state were trained to associate a particular auditory tone CS with unpleasantly-intense salt solution as UCS (triple the saltiness of sea-water), and a different CS with a pleasant sucrose solution as UCS; a third control CS meant nothing [12]. Neuronal firing was recorded in the rat's ventral pallidum during training. Only the CS for sucrose elicited high levels of firing. Then a physiological state of sodium depletion was induced overnight by injections of hormone-stimulating drugs (DOCA and furosemide) to induce a relatively sudden salt appetite. The rats had never experienced salt appetite before so the physiological state was new to them, and they were not allowed to taste any salt again until after the CS tests. In the new state, the CS tones were each presented a number of times by themselves, while mesocorticolimbic neural activations in their ventral pallidum were recorded. The CSs were presented by themselves (in extinction) so that no new experiences of the UCS tastes could influence the computation of CS incentive values.

**Figure 2:**
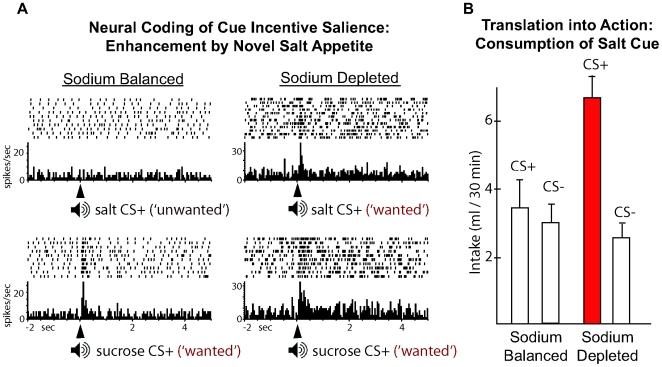
Natural salt appetite dynamically enhances incentive salience of a salt CS. Neural coding of CS ‘wanting’ illustrated by firing in ventral pallidum neurons (A). Ordinarily neurons in ventral pallidum that code CS for rewards fire to onset of an auditory tone CS that previously predicted infusion of sucrose solution into the rat's mouth (right column) but not to a CS for intense salt solution (A). A novel salt appetite state causes the neurons to fire as vigorously to the CS for salt as to the CS for sucrose while responses to sucrose cues persist unchanged (A). From [12]. Translation of enhanced CS incentive salience into action during salt appetite (B). When measured behaviorally, a novel salt appetite state causes rats to avidly consume a specific solution containing a gustatory CS (bitter or sour) that previously was paired with intense salt. Ordinarily the rats would not prefer to consume either CS solution [10],[50]. In all cases, the rats had not yet retasted actual salt UCS when they showed new ‘wanting’ of the CS.

The crucial observation in the electrophysiological results was that in the new salt appetite state, the salt CS now elicited a high level of firing that was equal to or even higher than the sucrose CS in the salt appetite state [12]. The dynamic elevation in firing pattern to a salt CS (Figure 2) indicates that the change in physiological state produced a dynamic elevation of incentive salience value of the relevant previously-trained CS. This boost in CS incentive coding was quite specific: it did not apply to a control CS that predicted nothing, nor did it further boost firing to the CS for sucrose.

Our additive model (Eqn 3b) can best capture these results, presuming that the motivation valence of incentive salience ‘wanting’ reverses from negative to positive (similarly to how hedonic ‘liking’ for intense salt reverses from ‘disliking’ during the appetite state). The additive model of reward modulation (Eqn 3b) explains this phenomenon as follows. Unpleasantly-intense salt originally takes on a negative reward value, and its incentive salience is <0. However, under sodium depletion, incentive salience causes κ to take on a value far greater than 1, i.e., log *κ* can be a large positive value for a salt CS. This should shift the reward value for salt associations sufficiently upward, so that salt becomes positive-valued as opposed to negatively valued.

Alternatively, we note that neuronal firing can only change from low to high or high to low, since a neuron's firing rate can never go below zero. Taken at face value, ‘wanting’, if represented linearly in VP firing, might merely change from zero to high during a particular state, without reversing valence. ‘Liking’, in contrast, demonstrably reverses in valence from ‘disliked’ to ‘liked’, requiring a separate neuronal coding mechanism. If VP firing to a cue linearly reflected univalent changes in ‘wanting’ primarily, then our multiplicative model of (Eqn 3a) would suffice to account for the dynamic modulation of incentive salience for salt appetite, as well as for the drug-related cases described below. However, it seems possible in principle that a univalent neural change might still encode a bivalent psychological shift in ‘wanting’. Resolving this translation of univalent neural firing into bivalent psychological values is beyond our current scope, though we have discussed it at greater length in other papers [50],[51],[71].

We also note that hedonic ‘liking’ as well as incentive salience ‘wanting’ for salt UCS and CS would be enhanced during the sodium appetite [51],[71]. Still, the point of this experiment was to show that the CS revaluation could occur as predicted by our model without experiencing the new UCS ‘liking’ or ‘wanting’ values, because rats were not allowed to taste the salt UCS in the new state until after the crucial test with the CSs.

Is the elevated incentive salience actually translated into motivated *action*? Dynamic elevation of appetitive and consummatory behaviors may additionally be observed if a CS is provided that supports a particular action, such as consumption behavior (Figure 2). For example if a gustatory CS is used in an experiment similar to above (such as a bitter or sour liquid as CS label, learned by being mixed with saltiness as UCS), then later the CS for salt (e.g., pure bitter solution or pure sour solution) by itself is becomes ingested during a salt appetite state, even if no salt is then available [10],[50],[70]. Previous studies in our lab and earlier ones by Fudim, and by Rescorla and Freberg, showed that rats sought out and selectively consumed a sour, bitter or other pure CS label that once was associated with a salt UCS, even if the CS taste solution was presented by itself without actual salt in the novel appetite test, and even if the rat had never yet tasted salt during the sodium depletion state (Figure 2). Such cases illustrate how natural appetite states can dynamically modulate incentive salience for a previously learned CS, guiding actions to ‘wanted’ targets, even before relearning any new information about its UCS.

### Effects of addictive drugs and mesolimbic sensitization on incentive salience

A special case of incentive salience modulation is incentive-sensitization: this occurs when drugs in the brain sensitize mesolimbic dopamine-related systems, and similar but temporary elevation of ‘wanting’ can be produced by directly injecting amphetamine before a test [8], [13], [72]–[74]. We capitalized on these drug-induced elevations in incentive salience to test the addiction-related predictions of (Eqn 3a) for enhancing cue-triggered wanting’ [8].

#### Test 2: addictive drugs activate and sensitize dopamine-related ‘wanting’

**Figure 3:**
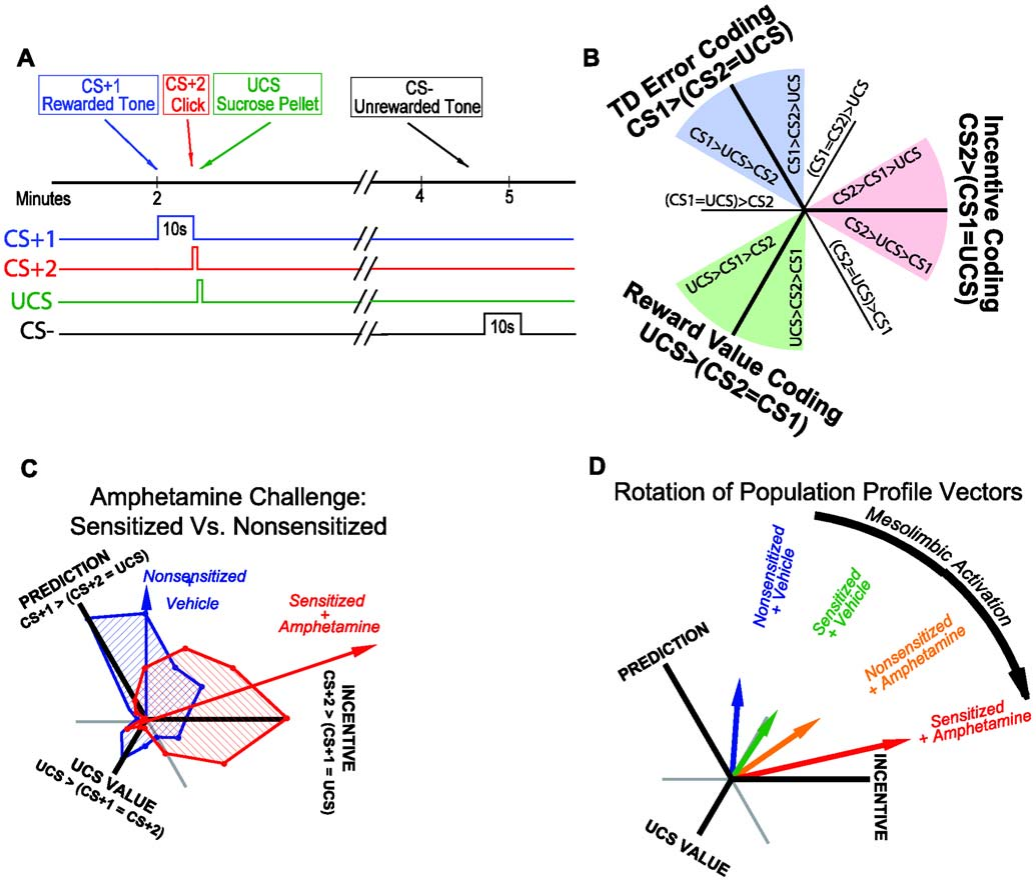
Selective amplification of CS incentive salience (not CS prediction or UCS hedonic impact) by transient amphetamine intoxication and more permanent drug sensitization. Experimental design of the serial CS1/CS2/UCS procedure, and effects of sensitization and amphetamine on neuronal firing profiles in ventral pallidum (A). The relative rank-ordering of neuronal responses to CS1/CS2/UCS is defined as the “profile” of a neuron; it can be represented mathematically as the angle of a vector in a two dimensional space, where the two axes represent two orthogonal contrasts formed from the three responses (B). The computation is such that this angular value indexing a response profile exists in a continuum which 1) exhausts all possible firing patterns (i.e., relative orders in firing rates to these three types of stimuli); and 2) guarantees that nearby values represents similar firing patterns. Temporal difference error-coding implies maximal response to CS1 which has the greatest prediction, whereas value-coding implies maximal firing to UCS which has the highest hedonic value. By contrast, incentive-coding implies maximal firing to CS2 that has the greatest motivational impact as it immediately precedes the actual reward. The data panel shows firing in control condition contrasted to the combination of amphetamine plus sensitization (C). The summary arrow panel shows the averaged neuronal response for each group of rats, illustrating the additive increments produced by sensitization, amphetamine and combination of both (D). Data from [13].

In studies to tease apart ‘wanting’ from ‘liking’ and from learned predictions of reward, we used a simple, serial paradigm containing two CSs that predicted a sucrose UCS [13],[75]. This serial CS paradigm helps separate the moment of maximal learned prediction (triggered by the first CS1) from the moment of maximal ‘wanting’ (triggered by the second CS2 that is temporally closer to a terminal sugary reward UCS), from the moment of maximum ‘liking’ (triggered by the sucrose UCS itself): thus, the full series was CS1→CS2→UCS (Figure 3).

After training in that serial association, some rats were sensitized by repeated binge-type doses of amphetamine [13]. Then, after a 1-month incubation period that allowed sensitization changes in brain mesolimbic systems to grow, we recorded neuronal firing in ventral pallidum to presentations of the CS1, CS2, and UCS reward stimuli. Those tests were conducted both in the absence and in the presence of an acute dose of amphetamine, on different days (see Figure 3 for the experimental design and timeline).

Note that the first CS (i.e., CS1) predicts all following stimuli, and because of temporal discounting, their magnitude will be in descending order: *V_1_*<*V_2_*<*r*, since UCS (when treated as a point reward) would be highest right when it is delivered. A pure TD value-coding model therefore predicts that neuronal coding should follow the same ordering, with activation to UCS being the largest. However, according to a pure TD model, the sensitization or administration of amphetamine should not immediately enhance cached CS values until after further training (relearning) with prediction errors from a potentially greater UCS after sensitization. Only with relearning (i.e., post-sensitization learning about surprising UCS) could the temporal difference prediction error signal *δ* “reboost” incentive salience attributions to the memory representation of the prior conditioned stimuli. Such reboosting based purely on associative learning requires re-experiencing the UCS under the sensitized (or otherwise revalued) condition.

By contrast, our gain-control *κ* model of incentive salience (Eqn 3) posits that any mesolimbic activation by psychostimulant sensitization or by acute amphetamine administration will immediately modulate the neuronal coding of a signal that carries high incentive salience for a previously learned CS. In particular, with respect to ordering of magnitudes, our model anticipates that CS2 should receive greater motivational impact than CS1, because it is closer in time to UCS and therefore most ‘wanted’ among CSs. Higher incentive salience of the CS2 was confirmed in the experiment by videoanalyses that showed motivated approach behaviors (nosepokes into the dish that delivered sucrose) were much higher during the CS2 than the CS1. Our prediction arises because in the serial Pavlovian conditioning paradigm, *r_1_* = 0 and *r_2_* = *r*, and Eqn (3a) indicates that 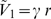, 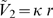. The temporal discount factor *γ*<1 and sensitization manipulation produces *κ*>1, thus 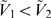.

In other words, because CS2 is the target of greatest incentive salience, its neural signal should be most potently enhanced by neural sensitization or by acute amphetamine administration that activates brain mesolimbic dopamine systems.

### Profile analysis supports incentive salience hypotheses for limbic firing

To compare these neuronal coding formulations against our model for incentive salience, we developed an analytic technique called Profile Analysis to assess neuronal responses to CS1, CS2 and UCS [13],[76].

Profile Analysis creates a quantitative index comparing the ordering of the magnitudes of a neuron's firing rates to the three stimuli, CS1, CS2, and UCS (Figure 3). The profile for each unit is defined as a vector in a two dimensional “profile space”. The direction of this vector reflects the rank-ordering of each neuron's firing rate responses to CS1, CS2 and UCS, while the magnitude of the vector reflects the degree to which the intensity of response to one stimulus dominates the responses to others). Inhibition of neuronal firing to a particular stimulus pulls coding vectors in opposite direction from excitations. All possible firing profiles are represented on a continuum of circular scale (360°), with nearby directions (angles) representing similar neuronal firing profiles. The profile analysis is performed on each individual neuron, and subsequently aggregated to obtain the entire neuronal population response.

More formally, let us denote each neuron's firing rate to CS1, CS2, and UCS (after normalizing to baseline) as *x*, *y*, *z* respectively [77]. The relative rank-ordering of these three numbers according to their magnitude represents the “profile” of a neuron's responses to the stimuli, and it can be represented mathematically as a vector in a two dimensional space. For each neuron we construct a two-dimensional unit vector (*u*,*ν*)from the three numbers *x*, *y*, *z*, such that they (i.e., the profile-representing unit vectors) are “equally spaced” in the projected two-dimensional subspace orthogonal to the direction [1,1,1]:
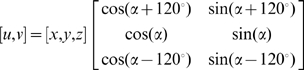
(6)where the “anchoring” parameter *α* can be chosen arbitrarily. For simplicity, we chose *α* = 0; in this case,
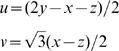
(7)The components of the profile vector [*u*,*v*] thus computed, according to Eqn. (6) in general and Eqn. (7) in particular, capture the two orthogonal contrasts formed among the three dependent variables *x*, *y*, *z*, such that any other contrast is a rotation in the two-dimensional space. This vector's magnitude
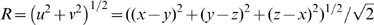
(8)represents the extent to which the neuron's firing rates, *x*, *y*, *z*, are differentially modulated by the three types of stimuli (CS1, CS2, and UCS), or in other words, it represents the variance of responses across the stimuli. The vector's direction
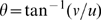
(9)reflects the type of rank-ordering of the magnitudes of these firing rates. This procedure is both exhaustive (i.e., all neurons can be characterized) and faithful (i.e., the distance between the angles is monotonically associated with the magnitude of difference in two profiles).

Of particular interest to this study are the regions corresponding to response dominance by a particular stimulus (Figure 3). The region spanning 60° to 180° is where CS1 dominates the response profile and represents neurons that are responsive to the CS1 cue which carries the most predictive information about subsequent stimuli. This is designated as the prediction or “TD error-coding” area. The region spanning −60° to 60° represents dominant neural firing to CS2, which occurs at moment of highest motivation excitement, and we denote it as the motivational or “incentive-coding” area. Finally, the region spanning 180° to 300° represents dominance by the reward itself (UCS) and it is designated the hedonic or “value-coding” area. Strictly speaking, our incentive salience theory predicts CS2>CS1>UCS for incentive-coding neurons, whereas if neurons obey a TD learning model, one predicts that the relative ordering of the magnitude of responses to the three stimuli after learning is CS1>CS2>UCS for error-coding neurons and UCS>CS2>CS1 for value-coding neurons. The rationale is similar to a method proposed by one of us earlier (called “Locus Analysis”) to characterize neurons in the primary motor cortex [78].

### Incentive shifts in neural profiles after amphetamine administration or drug sensitization

**Figure 4:**
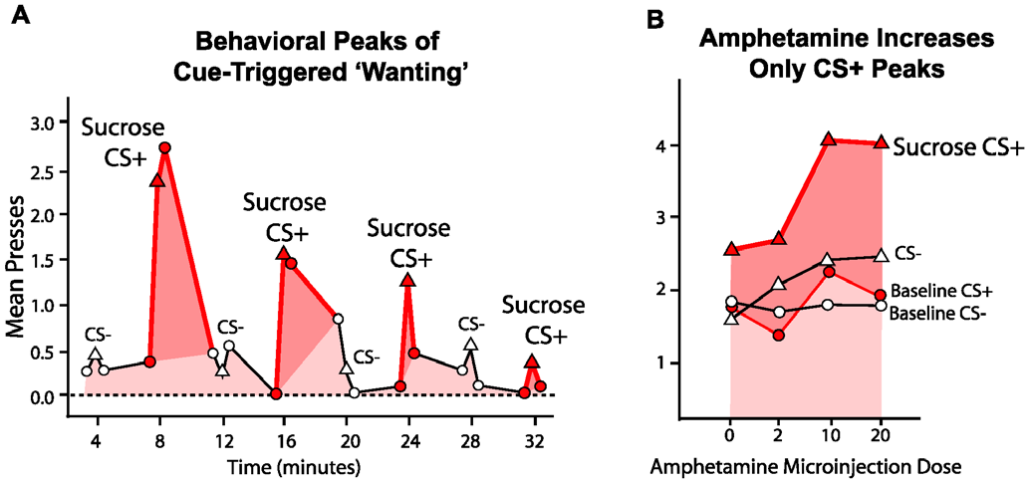
Behavioral confirmation of dynamic amplification of cue-triggered by amphetamine activation of mesolimbic systems. Transient ‘wanting’ comes and goes with the cue (A). Amphetamine microinjection in nucleus accumbens dynamically magnifies ‘wanting’ for sugar reward – but only in presence of reward cue (CS+). Cognitive expectations and ordinary wanting are not altered (reflected in baseline lever pressing in absence of cue and during irrelevant cue, CS−) (B). From [72].

The results of the amphetamine and sensitization experiment revealed that VP neurons ordinarily signalled best the prediction value of a CS, responding maximally to CS1 (Figure 3). Thus, in the normal state, these limbic circuits reflect the standard prediction error model. However, mesolimbic activation or sensitization changed this profile by enhancing only incentive salience signals to the CS2, at the expense of the signal for CS1 (and without altering UCS signal) [13] (Figure 4).

The incentive shift toward CS2 was even greater for the combination of sensitization plus amphetamine administration at the time of test. The effects of the various mesolimbic dopaminergic activations can be visualized as the rotation of the Population Profile Vectors (Figure 4). Thus, it was concluded that while VP neurons in control animals (after training) tend to follow a TD error coding profile, mesolimbic dopaminergic activation causes the neuronal response profiles to shift towards encoding incentive salience. This shift corresponds to our motivational transform computation model, and to the idea that mesolimbic stimulations enhanced *κ*.

Crucially, for showing the dynamic nature of the incentive increase, we note that the enhancements of neural firing to CS2 produced by amphetamine and by drug sensitization were evident right away on the very first presentations of the CS2 in the new sensitization and/or amphetamine state. That is, as predicted by our *κ* model, the incentive value of the CS2 was dynamically increased without need of re-learning about CS-UCS association, and without additional pairings with the UCS in the transformed state [13].

Does amphetamine or sensitization of incentive salience translate into behavioral ‘wanting’ too? In previous studies using a rigorous behavioral test of cue-triggered ‘wanting’ (based on a Pavlovian-Instrumental Transfer design or PIT), we and others have confirmed that acute amphetamine administration and/or prior drug sensitization both enhance peaks of cue-triggered ‘wanting’ for sucrose reward (Figure 4). In PIT, the phasic peaks of cue-triggered ‘wanting’ are manifest as a burst of pressing by the rat on a lever that previously earned sucrose reward: these peaks were dynamically enhanced by microinjection of amphetamine directly into the nucleus accumbens, or by sensitizing drug binges given weeks earlier [72],[73] (Figure 4). The ‘wanting’ enhancements occurred even on the first presentations of the CS in the new physiological states of mesolimbic activation, just as in the neural firing experiments above (Figure 4) [72],[73]. And the elevations came and went with the coming and going of the physical CS+ stimulus, which lasted about 30 sec each. Such dynamic enhancement of CS incentive salience is also consistent with other behavioral demonstrations of incentive motivation enhancement by pharmacological dopamine activation or by psychostimulant-induced neural sensitization [72],[73],[79],[80], even in the absence of additional learning [81]–[83]. Our conclusion is also compatible with other behavioral evidence that the most predictive CS can be dissociated from the most ‘wanted’ CS [84],[85]. Thus it seems safe to conclude that dynamic increases in incentive salience are expressed in behavior as well as in neural activation [15], [86]–[90].

## Discussion

As posited by our computational model (Eqns 3, 3a, 3b), dynamic enhancements of CS incentive salience can be empirically observed in both neural and behavioral measures of cue-triggered ‘wanting’. Enhancements were caused by relevant physiological changes, such as natural salt appetite, and addiction-related amphetamine intoxication and long term sensitization that modulate brain mesolimbic systems involving dopamine. All of these physiological manipulations revealed dynamic modulation of CS-triggered ‘wanting’ as posited by our model (Eqns 3; 3a; 3b).

### Experimental caveats

It is important to acknowledge that each experiment above is only an imperfect test of the model. The VP sensitization and amphetamine experiments hinged on the assumption that our sequential Pavlovian design decoupled the maximal predictive impact of CS1 from the maximal incentive impact of CS2. If the assumption were false, the conclusion would be weakened. Likewise, the salt CS study failed to cleanly separate ‘wanting’ from hedonic ‘liking’, because both were increased together during a natural sodium appetite. However, each experiment also carries strengths to counter these weaknesses. The sensitization-amphetamine experiment cleanly dissociated ‘wanting’ from ‘liking’ because the dopamine-based activations enhanced ‘wanting’ without at all enhancing sucrose ‘liking’. The salt experiment cleanly dissociated incentive ‘wanting’ from cached predictions gained by previous learning, without requiring serial CSs, because the previously learned CS value was negative and was dynamically reversed into positive valence at test by a natural specific appetite. Thus some confidence is gained by noting that our conclusions rest on the entire body of evidence rather than on any single experimental result.

### Forms of ‘wanting’ modulation

Comparing versions (Eqns 3a and 3b), we note that Eqn (3a) could be a standard way to express the generic model (Eqn 3) as a multiplicative gain-control mechanism, which generally applies to all univalent cases of modulation. These include enhancements of ‘wanting’ by amphetamine activation of mesolimbic dopamine systems and by permanent sensitization of mesolimbic systems, as shown here, and would also apply to natural cases such as a palatable food becoming more valuable in hunger. It would similarly apply to univalent downshifts in incentive value from good to less good or neutral (e.g., physiological satiety or dopamine suppression). Further, Eqn (3a) would also apply to changes in aversiveness from bad to worse (or vice versa) for fear, disgust or other negative evaluations that involve a negative version of motivation salience, such as changes in active fear or in psychotic paranoia produced by dopamine-blocking drugs or by psychological mood manipulations [53],[54],[91] (Figure 1).

Alternatively, Eqn (3b) expresses the generic model as an additive version, which is intended to account for special cases where incentive value actually reverses valence between positive and negative poles. Those include reversal of incentive salience for intense salt from negatively ‘unwanted’ to positively ‘wanted’ during salt appetite, and would also include flips from ‘wanted’ to ‘unwanted’ such as when a sucrose taste is converted from good to bad by aversion conditioning (i.e., pairing as CS with nausea as UCS), or flips between desire and dread [53],[90].

#### Comparing incentive salience model to standard reinforcement learning models

How does our model (Eqns 3; 3a; 3b) contrast to a cached-value TD model or to a flexible tree-search model involving a cognitive state space? According to a standard TD reinforcement model, optimal policy is invariant (i.e., remains unchanged) if the entire vector of primary rewards in every state is subject to the same affine transform (r−>ar+b, a>0) with fixed a,b. Cached values are relatively stable, and able to produce the same optimal behavior across a wide range of homeostatic/motivational states. But they are not able to dynamically modulate after a shift without further retraining.

The dynamic situations here, however, were very different and not amenable to a stable solution strategy, as the motivational revaluation occurred without the individual having had a chance to relearn. The cached value-function of each state (CSs) would not have been adjusted until after the next encounter with primary reward (UCS); the UCS itself would be immediately modulated by the physiological shift but would still need to be presented to effect a re-evaluation of CS.

More flexible are tree-search models, sometimes called model-based systems because they model or map the world in a way that explicitly represents goal values and relationships in a nested tree or similar recursive structure [14],[17],[21],[22],[39],[40],[92]. Psychological counterparts include cognitive maps of goal outcomes, with values obtained by episodic memories of experience with those goals in states similar to current conditions, and understanding of act-outcome relationships needed to obtain those goals [17], [18], [89], [92]–[94]. Still, some model-based formulations and psychological counterparts are constrained by whether the tree contains sufficient information to compute a new value in a state, which for some systems may depend on whether the goal has ever been tasted before while in a similar state [17],[18]. For example, as Dickinson and Balleine put it in describing the behavior of rats when guided by cognitive act-outcome relations involving hedonically positive memories of a sucrose goal they used to work for, but which had subsequently been devalued by taste-aversion conditioning: “we gave (some of) our rats the opportunity to taste the sugar after they had acquired a latent aversion to it. This re-tasting had a profound effect when we subsequently tested their desire to search for the sugar water by lever pressing. In the absence of the re-tasting, they behaved as though they were ignorant of their aversion…, whereas those that had re-tasted the sugar water showed little propensity to seek it out again (the devalued sugar water)” (p. 103) [17]. That is, the rat's cognitive system needed to re-taste the sugar in order to know that the former reward had become unpleasant and was no longer a positive goal. To the extent that computational models aim to capture real psychological cognition, retasting may remain an important feature of many model-based systems [17],[18],[21],[22],[28],[39],[40],[44].

Of course an empirical need for retasting by rat brains (or human brains) does not necessarily imply that all model-based RL computational mechanisms necessarily require retasting. Recently, Daw, Niv and Dayan have explicitly proposed an alternative tree model that can update without needing to retaste, using feedforward recalculation of goal value in a full look-ahead tree even before the goal is experienced [39],[95]. Such a model might be able to accomplish revaluations of CS value prior to UCS retasting such as those demonstrated in our experiments.

Yet differences remain between our Pavlovian-guided incentive salience model (mediated by mesolimbic brain circuits) and a cognitive map or full look-ahead tree model (plausibly mediated by cortical brain circuits) [39],[95]. One difference is that attribution of incentive salience to a CS makes the cue itself become motivationally ‘wanted’, beyond being a signal or trigger to ‘want’ the UCS goal [84],[85]. Another difference is that mesolimbic circuits computing *κ* modulate only the primary reward motivation and not state-values or state-action values. Loosely speaking, our model (Eqns 3; 3a; 3b) is analogous to one-step look-ahead in a model-based (tree-search) approach. Consider that cue-triggered ‘wanting’ shoots up upon presentation of a CS, but importantly, also goes down again nearly as soon as the CS is taken away. Coupling to CS is evident in behavioral PIT experiments, where lever-pressing peaks fade away as soon as the CS is removed (see figure 4) – even though the salt appetite, dopamine drug, or sensitization state that enhanced the cue's motivation-eliciting power persist. Neuronal VP firing peaks are even shorter, and linked to the onset of the CS presentation, and then typically decay within a second. Neither baseline levels of lever pressing rates nor neuronal firing rates were reliably enhanced at moments in between cues.

A full tree-model is usually thought to have an advantage of providing a stable cognitive map of declarative goals and available actions within the tree's representation of the world. Having once successfully recomputed the goal in advance of retasting the UCS, then, it may seem odd that a full look-ahead tree model should immediately abandon the goal value again as soon as the CS disappears, and to repeat the cycle each time the CS comes or goes. Yet to accommodate our data, some such transient and repetitive adjustment of a full-tree model would be required.

Transience, on the other hand, is quite typical of motivational states. In particular, the incentive salience mechanism is especially compatible with transient peaks in ‘wanting’ being tied to CS presence because the Bindra-Toates rules of Pavlovian motivation specify that a synergy exists between CS presence and current mesolimbic state, which controls the intensity of motivation at each moment [1]–[5],[8],[96]. The physical presence of a Pavlovian CS is a crucial factor in generating incentive salience, and a sporadic CS can lead to up-and-down changes in ‘wanting’. This synergy feature is precisely why hungers make their relevant cues much more powerful motivators, as well as why a food CS can trigger an increase in appetite for its UCS in a not particularly hungry person or a drug CS trigger relapse in an addict [2]–[5],[7],[8],[19],[89],[96],[97].

#### Multiple motivation-learning systems

We should acknowledge again that we do not suggest that incentive salience is the only computational form of goal-directed system operating in the brains of rats or people, any more than stimulus-stimulus Pavlovian associations are the only form of reward learning. Incentive salience ‘wanting’ is but one mechanism of motivation, occurring alongside others. For example, ample evidence described elsewhere indicates that ‘wanting’ (with quotation marks: incentive salience) exists alongside ordinary wanting (without quotation marks: cognitive predictions), which may plausibly be based on full look-ahead cognitive representations of expected goal values and their related act-outcome strategies to obtain those goals [3],[7],[14],[17],[18]. Ordinarily, wanting and ‘wanting’ act together to guide behavior toward the same goals, with incentive salience serving to add motivation ‘oomph’ to cognitive representations. But under some important conditions the two motivation mechanisms may diverge. For example, divergence can lead to ‘irrational wanting’ in addiction for a target that the individual does not cognitively want, nor predictively expect to be of high value [7]–[9]. Our current model may help to computationally capture the incentive salience limb of that divergence.

### Future challenges

Finally, we stress that our model (Eqns 3, 3a, 3b) is not meant as a finished model of incentive salience, but only is an incremental step towards more adequate computational models. Several important challenges remain. One challenge for a future incentive salience model is to better solve the specificity problem involved in the question of ‘what to want most’? That problem includes describing how specific types of *κ* (e.g., sodium appetite, hunger, drug sensitization) interact with specific CSs and their UCS rewards (e.g., salt, food, drugs) to determine the direction of maximal attribution of incentive salience toward a particular target. A related problem concerns the control of how sharply ‘wanting’ is focused by amygdala-related systems on one CS motivational magnet [48],[85], or on one UCS target in a winner-take-all fashion (as when an addict excessively ‘wants’ only drugs), or instead is spread somewhat over several targets (as when the addict also excessively ‘wants’ to gamble or engage in sex) [8]. Another challenge is to model the relation of incentive salience ‘reboosting’ (via incremental pairings of CS and UCS) to dynamic modulation (as shown here for a specific CS). A final challenge is to better capture the computational differences between Pavlovian-based ‘wanting’ described here versus tree-based cognitive goal systems and cached-based habit learning systems, and to better understand the conditions that determine whether those three systems cohere or diverge.

### Conclusion

To summarize, we have proposed a computational model of incentive salience as a motivational gating mechanism that dynamically responds to post-learning shifts in physiological states when encoding a relevant CS for reward. Our computation of incentive salience integrates a current change in physiological state with previously learned associations between a CS and its state-relevant UCS reward to generate ‘wanting’ in a dynamic and reversible fashion.

The computation of incentive salience outlined here implies that cue-triggered ‘wanting’ amounts to activating associations that exist between CS and UCS, and then dynamically recomputing motivational value based on current physiological state to generate the motivational magnet property of a reward cue [2],[3],[7],[8]. In natural appetites, like salt appetite or food hunger, the dynamic modulation is adaptive, and guides motivated behavior towards an appropriate incentive without need for stable experience-gained knowledge of the goal. In addicts, amplified motivation may maladaptively pull the addict like a magnet towards compulsively ‘wanted’ drugs, and so make it harder to escape from the addiction [8],[13],[98].
